# Editorial: Improving Early Detection and Risk Prediction in Heart Failure

**DOI:** 10.3389/fcvm.2022.930935

**Published:** 2022-05-18

**Authors:** Vinicius Tragante, Anna Pilbrow, Katrina Poppe

**Affiliations:** ^1^Department of Cardiology, Division of Heart and Lungs, University Medical Center Utrecht, Utrecht, Netherlands; ^2^Department of Medicine, Christchurch Heart Institute, University of Otago, Christchurch, New Zealand; ^3^Department of Medicine, University of Auckland, Auckland, New Zealand

**Keywords:** heart failure, systems medicine, prognostic models, diagnostic models, genomics

Heart failure (HF) is a debilitating and costly condition, characterized by repeated hospital admissions and high mortality. There is considerable heterogeneity in the underlying etiology, development and manifestation of the HF syndrome, with chronic conditions (including high blood pressure and diabetes), acute cardiac injury, genetics, and lifestyle interacting to influence the risk of developing clinical HF. As a consequence, best treatment practices are still relatively unknown and not tailored to HF subtypes. The goal of this Research Topic is to highlight the utility of systems medicine and omics approaches to refine HF sub-phenotyping beyond the common functional categories of HF with reduced, preserved or mid-range ejection fraction. Our hope is that bringing together diverse perspectives on this issue will result in a more global understanding of how these approaches can be used to improve early detection of HF and predict HF severity and prognosis.

In this special topic issue, we have compiled a wide variety of contributions from research groups working in this area. In total, 13 papers have been included, with a mixture of original research articles, reviews, a case study and a clinical trial protocol, that discuss incident HF, refinements to phenotyping HF, prognostic strategies (analytical and biomarker), and treatment.

Of these, two articles consider the identification of risk factors for incident HF. Sammani et al. compared text mining and machine learning approaches to screening electronic health records for people with unexplained left ventricular hypertrophy (ULVH). Text mining helped to identify a subset of patients with possible ULVH and reached a sensitivity of 0.78, whereas machine learning, with a specificity of 0.99, was recommended as a rule-out test. Gu et al. identified a reduced risk of incident HF after percutaneous coronary intervention in individuals taking ACEI/ARB in comparison to those on beta blockers.

Four articles discuss refinements to phenotyping HF or cardiomyopathy. In a literature meta-analysis of 9,491 HF patients from 9 studies, He et al. found that nearly a quarter of patients with HFrEF at discharge experienced improvement in left ventricular ejection fraction (EF) during on average 3.8 years of follow-up, and that the group with improved EF (HFimpEF) had substantially lower risk of all-cause mortality or cardiac hospitalization compared to those with HFrEF. Wang X. et al. report a case study in which a 68-year-old woman presented with frequent HF and shock, which was found due to tertiary adrenal insufficiency caused by long-term corticosteroid use, even though this is not considered a risk factor for cardiomyopathies. Topf et al. compared circulating levels of cardiac biomarkers (sST-2, GDF-15, suPAR, and HFABP), clinical and imaging factors of 51 patients with Takotsubo cardiomyopathy, 52 with ischemic cardiomyopathy and 65 with dilated cardiomyopathy. sST-2 was the best discriminator of the three phenotypes. Stojanovic et al. sought to test whether renalase, a potential new marker for myocardial ischaemia, would enhance identification of underlying ischaemic heart disease in people with chronic HF. Renalase was similarly predictive of ischaemic changes during an exercise stress test as BNP, and the authors suggest both biomarkers could be used to help identify patients with HF who have underlying ischaemic disease, particularly in patients with HFrEF.

There are also new prognostic strategies. Gao et al. used supervised machine learning to identify biomarkers with prognostic value for HFpEF and found a support vector machine approach outperformed a Cox regression model of similar predictors. In 104 patients with newly diagnosed idiopathic dilated cardiomyopathy, Xie et al. tested multiple machine learning methods to predict reverse remodeling and so help identify patients who may be resistant to optimal treatment. Discrimination analysis found that extreme gradient boosting, using markers such as cystatin C, right ventricular end-diastolic dimension and HDL-C, may help differentiate responders from non-responders. Yang et al. show better performance of an extreme learning machine Cox model in comparison with Lasso Cox and random survival forest models to predict the risk of worsening prognosis in patients with chronic HF, particularly as censoring ratios increased. Finally, Hu et al. used transcriptomics to identify a set of genes with altered expression in failing hearts compared to healthy donors. From those, a model using a 31 SNP genetic risk score, combined with traditional factors, demonstrated a 22-fold increased risk of mortality in individuals with a high composite risk compared to individuals with a low composite risk.

With a focus on identifying new markers of HF progression, Liang et al. present a mini review showing that hydration, measured by bioimpedance, associates with longer hospital stays and worse outcomes in acute and chronic HF, suggesting bioimpedance could improve the clinical assessment of acute HF. In a prospective study, Wang C. et al. found that the ratio of cystatin C to prealbumin was predictive of both cardiovascular and all-cause death, independent of established risk factors and NT-proBNP.

Also in this issue, Cho et al. present a study protocol to investigate the clinical efficacy and safety of rivaroxaban compared with warfarin in patients with chronic HF and atrial fibrillation. The expectation is that rivaroxaban will reduce myocardial injury and hemodynamic stress in this patient group, paving the way to new treatments.

Taken together, these papers highlight the diversity of systems medicine approaches in HF and provide insight into the many exciting avenues of research that continue to enhance our understanding of the HF syndrome. Beyond these approaches, we have identified key areas where systems medicine may accelerate improved early detection and risk prediction in HF. These include (i) appropriate consideration of ethnicity and other demographic factors to develop population-specific, personalized strategies for diagnosis and treatment; (ii) use of large, multi-modal datasets, with consideration of the pros and cons of including routinely collected administrative health data with clinical data, imaging, biomarker and genomic information to more accurately model HF risk and outcomes; and (iii) adjustment for the time-varying contribution of predictors through the life course of HF. For example, research that combines clinical and genomic information needs to consider how to equitably analyse the long-term cumulative effects of genomic risk factors with the short-term impact of clinical markers which are often measured at the time of an acute event. Systems medicine approaches are also suited to tackling complex outcomes beyond the “time to first event,” such as the burden of recurrent hospitalizations, which is more relevant to a chronic condition. The target of research then becomes how to reduce the burden of HF on health systems and families. Lastly, there is an urgent need for advanced systems-based methodologies, including nuanced modeling structures that go beyond standard linear regression, more refined and informed use of machine learning approaches, including for natural language processing, and better consideration of the stage of HF being researched (acute presentation or during stable follow-up, at the time of diagnosis or years after the onset of HF).

In summary, there is an unmet need for improved methods for diagnosis, prognosis, and management of HF. We believe that use of systems medicine and omics approaches in well-phenotyped cohorts may inform the mechanisms underlying the development and progression of HF and ultimately lead to more personalized medical monitoring and treatment, increasing patients' lifespan and quality of life.

## Author Contributions

VT, AP, and KP contributed equally to the writing of this manuscript. All authors contributed to the article and approved the submitted version.

## Funding

AP was supported by the New Zealand Heart Foundation 100 Fellowship (Grant No. 1910) and also KP was supported by the New Zealand Heart Foundation Hynds Senior Fellowship (Grant No. 1755).

## Conflict of Interest

The authors declare that the research was conducted in the absence of any commercial or financial relationships that could be construed as a potential conflict of interest.

## Publisher's Note

All claims expressed in this article are solely those of the authors and do not necessarily represent those of their affiliated organizations, or those of the publisher, the editors and the reviewers. Any product that may be evaluated in this article, or claim that may be made by its manufacturer, is not guaranteed or endorsed by the publisher.

